# Harnessing Malaria Surveillance Data for Transformative Malaria Control and Elimination

**DOI:** 10.4269/ajtmh.24-0817

**Published:** 2025-01-07

**Authors:** Yazoumé Yé, Debra Prosnitz

**Affiliations:** ^1^ CESMEL Health, Bowie, Maryland;; ^2^ CDC Foundation, California

The momentum for data-driven decision-making, particularly for disease surveillance, including for malaria, is greater than ever. Surveillance data that is appropriately collected, analyzed, visualized, and used can effectively guide context-specific and responsive efforts for disease burden reduction and elimination. This journal supplement which follows the prior supplement, “*Malaria Surveillance as a Core Intervention,*”^1^ published in 2023, focuses on “***Surveillance Data for Decision-Making in Global Health: Enhancing Analysis, Integration, and Action***.” The eight articles in this supplement highlight the importance of ensuring high-quality and comprehensive data and explore the use of vector control, molecular, and genomic information to optimize malaria interventions for reduction of the disease burden.

Ye et al.^2^ assessed data management practices and the quality of malaria routine surveillance data in Madagascar, revealing several data–quality gaps, particularly data incompleteness and reporting inaccuracy.^2^ The authors recommend a regular review of malaria data quality at the service delivery point using a standard tool such as the Malaria Routine Data Quality Assessment Tool^3^ to improve the reliability of the data and create a culture of information use at all levels. Specifically exploring reporting accuracy, a data quality challenge driven mainly by health care providers’ behavior, Karemere et al.^4^ tested the introduction of automated rapid diagnostic test readers, which can read and report malaria test results automatically. These devices have great potential for reducing human errors in malaria surveillance data; however, there are technological and logistical challenges to address for them to be effective, especially at remote health service delivery points, where reporting inaccuracy can be common. Another key data quality challenge in malaria surveillance is an incomplete picture of the disease burden because private sector data are not consistently included in national health information systems. Berlin et al.^5^ argue that integrating private sector data into national surveillance systems through mapping providers, incorporating them into digital registries, and enforcing standardized reporting can improve the accuracy of data. Experiences from the Greater Mekong and sub-Saharan Africa suggest that the integration of private sector data will improve data-driven decision-making for malaria programming.

Ensuring high-quality malaria surveillance data helps provide information for accurate decision-making; however, leveraging and integrating other non-traditional sources of malaria-related data may provide additional insights enabling more focused malaria control efforts, particularly at sub-national levels. Along these lines, Burnett et al.^6^ discuss the need to integrate routine health data and entomological and program vector control data to evaluate vector control interventions comprehensively. The authors provide practical guidance on evaluation design and recommend key variables, data sources, and methods to address data quality challenges to increase frequency, rigor, and use of routine data to inform decision-making for vector control. Golumbeanu et al.^7^ discuss the increasing importance of molecular and genomic assays in studying malaria parasites and their potential to inform decision-making for policy and programs. Molecular and genomic surveillance could further support malaria elimination efforts in low-transmission settings if they are effectively integrated into traditional epidemiological surveillance and if laboratory capacity is strengthened at the local level. Further exploring ways to improve the quality, access, and use of malaria surveillance data using digital technology, Stratil et al.^8^ assessed the rollout of the Integrated Malaria Information Storage System (iMISS) platform with the use of electronic reporting from health facilities in Mozambique. They concluded that iMISS improved the accuracy and reporting rate of data despite several challenges with system maintenance and internet connectivity.^9^

Despite the remaining system and technological challenges discussed in these articles, there has been a significant improvement in the quality of malaria surveillance data and rising interest in making these data the backbone and drivers of success in malaria control and elimination efforts. The interest is manifest in the World Health Organization (WHO) Reference Manual for Malaria Surveillance, Monitoring & Evaluation,^10^ and also with several initiatives, which have streamlined the use of malaria surveillance data and other malaria-related data sources. These initiatives, led by the WHO Global Malaria Programme, include two WHO programs: Sub-National Tailoring and the Country Malaria Data Repository for Malaria Control, both part of the High Burden to High Impact approach.^11^ Moving away from one-size fits all, the Sub-National Tailoring approach advocates and provides guidance for using granular data at the subnational level to define a package of interventions that fits specific patterns and trends of malaria transmission risk. This will ensure optimal use of resources for greater impact. The Country Malaria Data Repository for Malaria Control facilitates the use of data. These platforms integrate surveillance data, research findings, and programmatic data, enabling comprehensive analysis and interpretation to guide malaria programs at all levels of the healthcare system.

The improvement of malaria surveillance data presents a great opportunity for evaluating the effectiveness of malaria programs at national and subnational levels; this is especially salient for new malaria interventions. With the recent rollout of RTSS and R21/Matrix malaria vaccines, national malaria programs should leverage improved malaria surveillance data to monitor and assess vaccine impact.^12^ This is critical, because vaccine supply is limited and must be strategically allocated based on available evidence. Furthermore, countries should leverage a strengthened surveillance system with emerging digital technology to monitor drug efficacy and detect any signs of drug resistance leading to decreased efficacy of artemisinin-based combination therapies to treat malaria. To protect one of our best tools for controlling malaria, the WHO developed a strategy document to respond effectively to any threat of antimalarial drug resistance in Africa.^13^ The strategy recognizes the challenges of limited data availability and inconsistent drug resistance surveillance systems in Africa; therefore, the first of five pillars recommends that countries and the global malaria community strengthen surveillance of antimalarial drug efficacy for timely detection of and response to resistance.

Given the critical role malaria surveillance plays in control and elimination efforts, funding for surveillance remains insufficient, limiting the ability of countries to implement required actions to strengthen and sustain reliable surveillance systems. We hope the challenges and opportunities discussed in this supplement will serve as a call to action for increased funding for malaria surveillance – from point of service collection to analysis and interpretation of data – which will help countries adopt emerging technologies to sustain current gains and strive for further improvement.

## References

[1] RBM Partnership to End Malaria, Surveillance, Monitoring, and Evaluation Reference Group (SMERG), 2023. Surveillance as a core malaria intervention. Am J Trop Med Hyg 108 *(**Suppl 2**):* 1–37.

[2] Ye M, , 2025. Identifying strengths and gaps in data management and reporting through malaria routine data quality assessment: Results from two health regions in Madagascar. Am J Trop Med Hyg 112 *(**Suppl 1**):* 3–9. 10.4269/ajtmh.23-0224 PMC11720680 38266305

[3] MEASURE Evaluation, 2020. Malaria Routine Data Quality Assessment Tool: A Checklist to Assess the Quality of Malaria Program Data. Chapel Hill, NC: MEASURE Evaluation, University of North Carolina. Available at: https://www.measureevaluation.org/resources/publications/tl-20-85/. Accessed September 11, 2024.

[4] Karemere J, , 2025. Evaluating the implementation of automated malaria rapid diagnostic test readers in health facilities in the Democratic Republic of Congo: Process, challenges, and lessons learned. Am J Trop Med Hyg 112 *(**Suppl 1**):* 10–16. 10.4269/ajtmh.23-0670 PMC11720685 39437775

[5] Berlin E Lourenco C Poyer S, 2025. Surveillance for action: Operationalizing private sector surveillance and service delivery across the malaria transmission continuum. Am J Trop Med Hyg 112 *(**Suppl 1**):* 66–69. 10.4269/ajtmh.23-0447 PMC11720681 38653216

[6] Burnett SM, , 2025. Process and methodological considerations for observational analyses of vector control interventions in sub-Saharan Africa using routine malaria data. Am J Trop Med Hyg 112 *(**Suppl 1**):* 17–34. 10.4269/ajtmh.22-0757 PMC11720682 37604476

[7] Golumbeanu M Edi CAV Hetzel MW Koepfli C Nsanzabana C, 2025. Bridging the gap from molecular surveillance to programmatic decisions for malaria control and elimination. Am J Trop Med Hyg 112 *(**Suppl 1**):* 35–47. 38150733 10.4269/ajtmh.22-0749 PMC11720679

[8] Clinton Health Access Initiative, 2019. User Requirements Document– iMISS Integrated Malaria Information and Storage System. Available at: https://www.clintonhealthaccess.org/annual-report-2019/. Accessed December 7, 2024.

[9] Stratil AS, , 2025. Improving the quality and use of malaria surveillance data: Results from evaluating an integrated malaria information storage system at the health facility level in selected districts in Mozambique. Am J Trop Med Hyg 112 *(**Suppl 1**):* 59–65. 38266287 10.4269/ajtmh.23-0138 PMC11720678

[10] World Health Organization, 2018. Malaria Surveillance, Monitoring and Evaluation: A Reference Manual. Available at: https://iris.who.int/handle/10665/272284. Accessed December 7, 2024.

[11] World Health Organization (WHO) and RBM Partnership to End Malaria, 2019. High Burden to High Impact: A Targeted Malaria Response. Available at: https://reliefweb.int/report/world/high-burden-high-impact-targeted-malaria-response. Accessed December 7, 2024.

[12] Duffy PE Gorres JP Healy SA Fried M, 2024, Malaria vaccines: A new era of prevention and control. Nat Rev Microbiol 22: 756–772. 39025972 10.1038/s41579-024-01065-7

[13] World Health Organization (WHO), 2022. Strategy to Respond to Antimalarial Drug Resistance in Africa. Available at: https://www.who.int/publications/i/item/9789240060265. Accessed December 7, 2024.

